# Dissection of voltage-gated sodium channels in developing cochlear sensory epithelia

**DOI:** 10.1007/s13238-015-0157-1

**Published:** 2015-05-05

**Authors:** You Zhou, Fang-Hao Fang, Zhi-Rui Liu, Yong-Hua Ji

**Affiliations:** Laboratory of Neuropharmacology and Neurotoxicology, Shanghai University, Shanghai, 200436 China

**Dear Editor,**

Hair cells (HCs) clustered in cochlear sensory epithelia produce spontaneous spike and evoked action potentials during the developmental stage of auditory, which rely on many ion channels, such as Ca^2+^ channels, K^+^ channels and Na^+^ channels (Housley et al., 2006; Marcotti et al., 2003). As the dominant molecule acting in action potentials in most of excitable cells, Na^+^ channels (VGSCs) were deemed to be indispensable in the spontaneous and evoked action potentials of HCs (Marcotti et al., 2003). The inward Na^+^ currents recorded from hair cells, shorten the time to reach the action-potential threshold (Eckrich et al., 2012). However, little was known about the tissue- or developmental expression and the molecular structural information of Na^+^ channel subtype in hair cells, which extremely restricts the understanding of physiological mechanisms related to hearing development.

To examine whether there exist any novel sodium channel subtype in cochlear hair cells, the sensory epithelia locating in premature mouse cochlea was carefully dissected and used for the identification of VGSCs in cochlear hair cells. Three pairs of degenerate primers derived from conserved region of mammalian VGSCs (Table S2), as well as their invertebrate counterparts (from Drosophila, cockroach, housefly and mosquito), were used for detecting the homologous sequence of cochlear VGSCs. All the resultant sequences were matched to the known mammalian VGSC subtypes (Na_v_1.1–1.9, data not shown), indicating that no novel sodium channel subtype could be found in cochlea. Subtype-specific primers corresponding to conserved regions of Na_v_1.1–1.9 were designed and used to probe and quantify the abundance of VGSCs expressed in cochlea (Table S1). Analysis of these cDNA fragments ranging from 115 to 340 bp revealed that all the known VGSC subtypes could be detected in cochlea though in a less expression level than that of Ca_v_1.3 (Fig. 1A).

**Figure 1:**
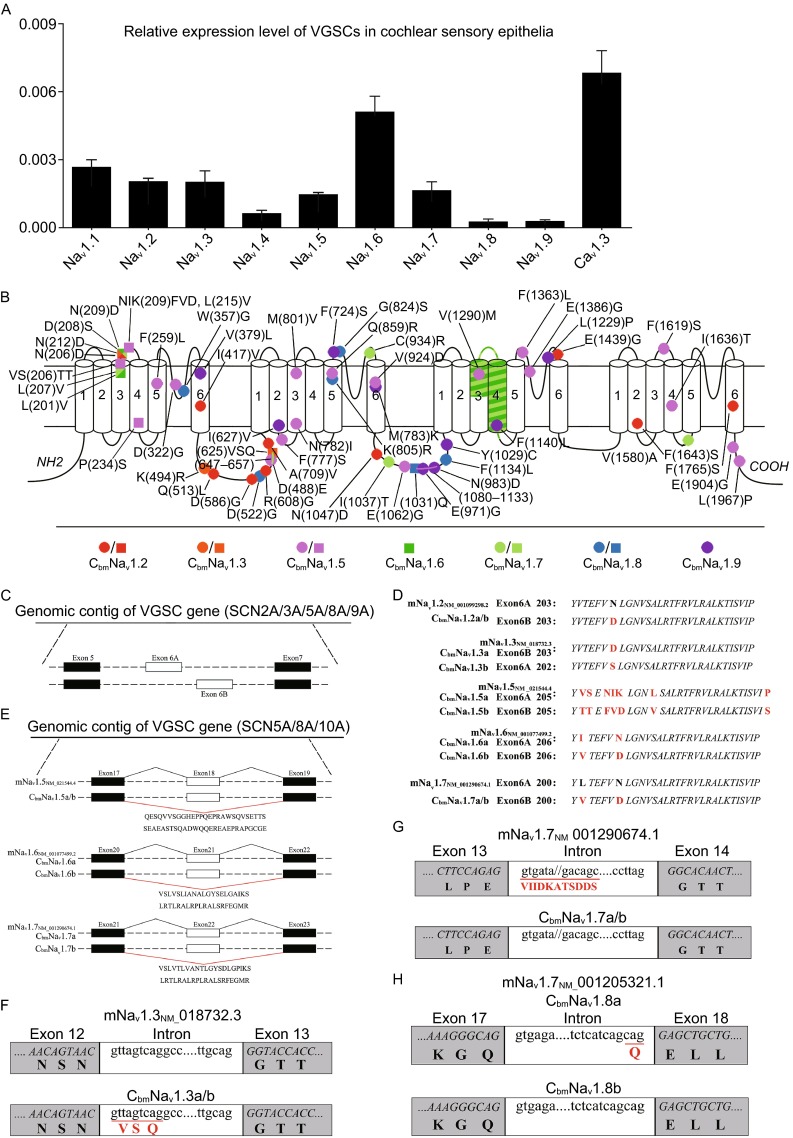
**Molecular characteristics of sodium channels expressed in cochlear sensory epithelia**. (A) Quantitative analysis of nine subtypes of sodium channels expressed in cochlear sensory epithelia. The mRNA encoding for TTX-R Na_v_ channels (mNa_v_1.5, mNa_v_1.8 and mNa_v_1.9) were detected of lower expression level, the mRNA copy numbers of TTX-S Na_v_ channels were shown at high level. (B) The schematic diagram of the sodium channel topology showed RNA editing and alternative splicing sites of C_bm_Na_v_s. RNA editing sites were illustrated with circles icon; and alternative splicing sites were highlighted with square icon. See Table 1 for details on these mutations. (C–H) Alternative splicing events occured in cochlear sodium channels

Co-expression of multiple Na^+^ channel isoforms has been described not only in neurons from the brain but also in primary sensory receptors of the mammalian cochlea. Na_v_1.7 has been suggested as the main carrier of I_Na_ in mouse inner hair cells because of the distinct biophysical and pharmacological properties (Marcotti et al., 2003). Both Na_v_1.1 and Na_v_1.6 were identified through immunohistochemistry on cell membrane of rat hair cells, while Na_v_1.2 was localized to the unmyelinated efferent axons and terminals (Eckrich et al., 2012). Na_v_1.6 with high expression in sensory epithelia is considered as the developmental regulator involved in the frequency of action potential activity and hair cell maturation. In this study, four sodium channels mentioned above were found with pronounced expression, providing further evidence of these subtypes in regulating action potential (Eckrich et al., 2012; Marcotti et al., 2003). Nevertheless, Na_v_1.4, Na_v_1.5, Na_v_1.8 and Na_v_1.9, were found to be expressed in relatively low levels in cochlear sensory epithelia (Fig. 1A).

To obtain a clear view on the structural features of cochlear VGSCs, specific primers were designed to clone the coding sequences (CDS) of all the VGSCs identified (Table S2). The sequences of cochlear Na_v_1.1 and Na_v_1.4 were identical with that of counterparts in other mouse tissues. The other seven full-length VGSCs or structural variants comprised 5298 and 6021 bp (Genebank number: KM373687-373700), encoding for 1765 and 2006 amino acids.

According to the generally acceptable nomenclature (Goldin et al., 2000), the cochlear VGSCs mentioned above were named as C_bm_Na_v_1.x, in which the subscript letter ‘bm’ indicates the originated tissue of cochlear VGSCs and ‘1.x’ indicates the corresponded mammalian Na_v_ counterparts. For instance, cochlear VGSC corresponding to Na_v_1.2 was named C_bm_Na_v_1.2. Hereinafter, alternative splicing or RNA-editing variants that belong to C_bm_Na_v_1.2 were named as C_bm_Na_v_1.2a, C_bm_Na_v_1.2b…, respectively. When mapping these sequences of C_bm_Na_v_1.x to the mouse genome, it was found that C_bm_Na_v_1.x were subjected to diverse point mutations, fragment insertions and deletions brought by the alternative splicing and RNA-editing events, most of which sit in functionally significant regions (Fig. 1B). All the editing sites and alternative splicing sites were double checked by pyrosequencing to remove any interference of RT-PCR errors. The sites location and nucleotide substitution patterns of all the alternative splicing and RNA-editing variants were shown in Table 1.

**Table 1:** Common and unique amino acid changes in fifteen C_bm_Na_v_ variants

	C_bm_Na_v_1.2	C_bm_Na_v_1.3	C_bm_Na_v_1.5	C_bm_Na_v_1.6	C_bm_Na_v_1.7	C_bm_Na_v_1.8	C_bm_Na_v_1.9
t-a			V924D^a^				M783K^abc^
t-g			D488E^b^			W357G^ab^	
t-c	V1580A^a^ F1765S^b^		F259L^a^ F777S^a^ F1363L^a^ I1636T^a^ F1619S^b^ L1967P^b^		C934R^ab^ I1037T^ab^ F1643S^ab^	F1134L^ab^	F724S^abc^ F1140L^b^ L1229P^b^
a-g	I417V^b^ D586G^b^ R608G^a^ N1047D^b^ E1439G^b^	K494R^a^	D322G^a^ Q859R^a^ M801V^b^ E1062G^b^ E1386G^b^ E1904G^b^			D522G^ab^ K805R^ab^	I627V^abc^ E971G^c^ N983D^c^ Y1029C^b^
a-t	Q513L^b^		N782I^a^				
g-a			V1290M^b^			G824S^ab^	
g-t							V379L^abc^
c-t	A709V^a^						
Substitution	N209D^ab^	D208S^b^	VS206TT^b^ NIK209 FVD^b^ L215V^b^ P234S^b^	I207V^b^ N212D^b^	L201V^ab^ N206D^ab^		
Deletion			1080–1133^ab^	1272–1312^b^	1266–1306^b^ 647–657^ab^		
Insertion		625VSQ^ab^				1031Q^b^	

Amino acid sequences of these variants (a, b and c represent different variant of each VGSC subtype) were compared with the genome sequence of corresponding subtypes. Nucleotide bases of RNA editing events that correspond to these in the mNa_v_ genomic sequence were in bold. Alternative splicing events included exons mutual repulsion resulting amino acid changes, exons kipping resulting amino acid deletion, alternative 3′ splicing and alternatives 5′ splicing resulting amino acid deletion and insertion

Two variants of C_bm_Na_v_1.2 differed by a single exon 6 exhibited the same alternative splicing site (Fig. 1C and 1D), which maybe resulting in functional enhancement as observed in hNa_v_1.2adu (Xu et al., 2007). Likewise, cochlear hair cells expressing alternatively splicing variant of C_bm_Na_v_1.2 may display more excitable ability. R608G, A709V, Q513L and D586G identified in C_bm_Na_v_1.2 locating at intracellular loop between DI and DII were potentially adjacent to the phosphorylation sites may greatly influence the channel activity (Chahine and O’Leary, 2014). RNA-editing sites I417V, E1439G and F1765S were located in extracellular loop between S5 and S6 of C_bm_Na_v_1.2, relating to ionic permeability and selectivity (Fig. S1).

Na_v_1.3 is mainly expressed in CNS during the early development stage (Hains et al., 2003). In rNa_v_1.3 or hNa_v_1.3, extensive alternative splicing sites, such as 12v1, 12v2 and 12v3 (rat), as well as 12v1, 12v2, 12v3 and 12v4 (human) in DI-II intracellular loop were identified to be important for channel kinetics (Thimmapaya et al., 2005). Alternative splicing site of 12v2 was also detected in C_bm_Na_v_1.3 (Fig. 1F). A missense mutation, K494R, was found in intracellular loop between DI and DII in both variant C_bm_Na_v_1.3a and C_bm_Na_v_1.3b. Similar to rNa_v_1.2, a missense mutation D208S of C_bm_Na_v_1.3 at extracellular loop between S3 and S4 of the DI was caused by alternative splicing on Exon 6A or Exon 6B (Fig. 1C and 1D). All of these mutations might have substantial impact on phosphorylation and gating properties of the channel.

To date, seven alternative splicing variants of Na_v_1.5 including Na_v_1.5a (exon 18 deletion), Na_v_1.5b (exon 17 and exon 18 deletion), Na_v_1.5c (CAG inclusive variant), Na_v_1.5d (exon 17 splicing), Na_v_1.5e (exon 6 splicing), Na_v_1.5f (exon 24 deletion) and C-terminal splice variants, have been reported (Schroeter et al., 2010). According to the splicing pattern, distribution and expressive proportion, these variants showed diversities in different species and different tissues of same species. Of all the VGSC subtypes found in mouse cochlear sensory epithelia, C_bm_Na_v_1.5 was subjected to the most intensive RNA-editing modification, among which T-to-C and A-to-G were the two common nucleotide substitution patterns, containing six editing sites each. Surprisingly, C_bm_Na_v_1.5 was substantially modified by alternative splicing as well, resulting in two alternative splicing variants having significant deletion at functionally important regions of the channel: to have seven amino acid residues substitution at DI S3–S4 extracellular loop were found in C_bm_Na_v_1.5b. In addition, C_bm_Na_v_1.5a and C_bm_Na_v_1.5b lost the entire exon 18 at DI-DIII intracellular loop which contained 54 amino acid residues (Fig. 1E, Table 1). Notably, exon 18 deletion, encoding for 53 amino acids at the DII–DIII linker (residues 1078–1130 in hNa_v_1.5), was the first reported splice variant in the cardiac Na^+^ channel. This splicing variant showed a pronounced species-specific expression pattern in mammals (Blechschmidt et al., 2008).

Na_v_1.6 is widely expressed in both PNS and CNS (Caldwell et al., 2000). A variant of hNa_v_1.6 lost a part of S3 and the whole transmembrane S4 at DIII (Zubovic et al., 2012a). Alternative splicing sites previously found in rNa_v_1.6 and hNa_v_1.6 were also appeared in C_bm_Nav1.6b variants which appeared as a loss of exon 21, taking away partial sequence of DIIIS3 and the entire DIIIS4 (Fig. 1E). Our finding identified the same deletion in C_bm_Na_v_1.6b of mouse cochlea (Fig. 1E). Deletion of exon 21 caused the loss of voltage sensor S4, it may be a “fail-safe” mechanism that produces a non-functional truncated protein (Plummer et al., 1997).

Alternative splicing variants of Na_v_1.7 were found in dorsal root ganglia (DRG) neurons under neuropathic pain conditions of rats (Dib-Hajj et al., 2013). In this study, C_bm_Na_v_1.7a and C_bm_Na_v_1.7b were characterized by the existence of exon 6B, differing at two amino acids (201V, 206D) in the D1/S3–S4 linker comparing to mNa_v_1.7 (201L, 206N) (Fig. 1C and 1D). Previous study revealed that the variant containing exon 6B had slower kinetics of inactivation for negative potentials than that of the variant containing exon 6A (Chatelier et al., 2008; Jarecki et al., 2009). Meanwhile, exon 22 deletion was first observed in C_bm_Na_v_1.7b which was lacking partial sequence of DIIIS3 and the entire DIIIS4 (Fig. 1E), suggested that the truncated protein maybe bear a similar protection mechanism to Nav1.6 (Plummer et al., 1997; Zubovic et al., 2012b).

For C_bm_Na_v_1.8, it was found five RNA-editing sites located at the DI/DII pore region and intracellular loops. Nine novel RNA-editing sites were identified in two C_bm_Nav1.9 variant, eight of which were caused by the nucleotide changes of adenine to guanine (Table 1). The alternative splicing event caused a deletion of Glu at position 1031 of C_bm_Na_v_1.8b (Fig. 1H). Few cases have been approached as for the functional consequences of these mutations. Although several mutation sites in Na_v_1.8 and Na_v_1.9 variants were identified, whether these mutations may diversify the function of channels will still need further researches.

Groundbreakingly, all known subtypes of sodium channels were identified in mouse cochlear sensory epithelia before hearing onset, strongly indicating the indispensible role in hearing development and formation. Moreover, some structural variations of C_bm_Na_v_ were found, possibly endowing distinct functions in order to adapt to the physiologically distinct properties for the cochlear VGSCs. This work lays a foundation for further studies to understand the roles of diverse C_bm_Na_v_ variants in shaping action potentials in cochlea and regulating hearing development.

## Electronic supplementary material

Supplementary material 1 (PDF 348 kb)
