# Supplementary material for: Dissection of voltage-gated sodium channels in developing cochlear sensory epithelia
Source: Protein Cell. 2015 May 5;6(6):458–62. doi: 10.1007/s13238-015-0157-1 (PMC4444814; doi:10.1007/s13238-015-0157-1)
Supplement: Supplementary file 1 — Supplementary material 1 (PDF 348 kb) [file 13238_2015_157_MOESM1_ESM.pdf]

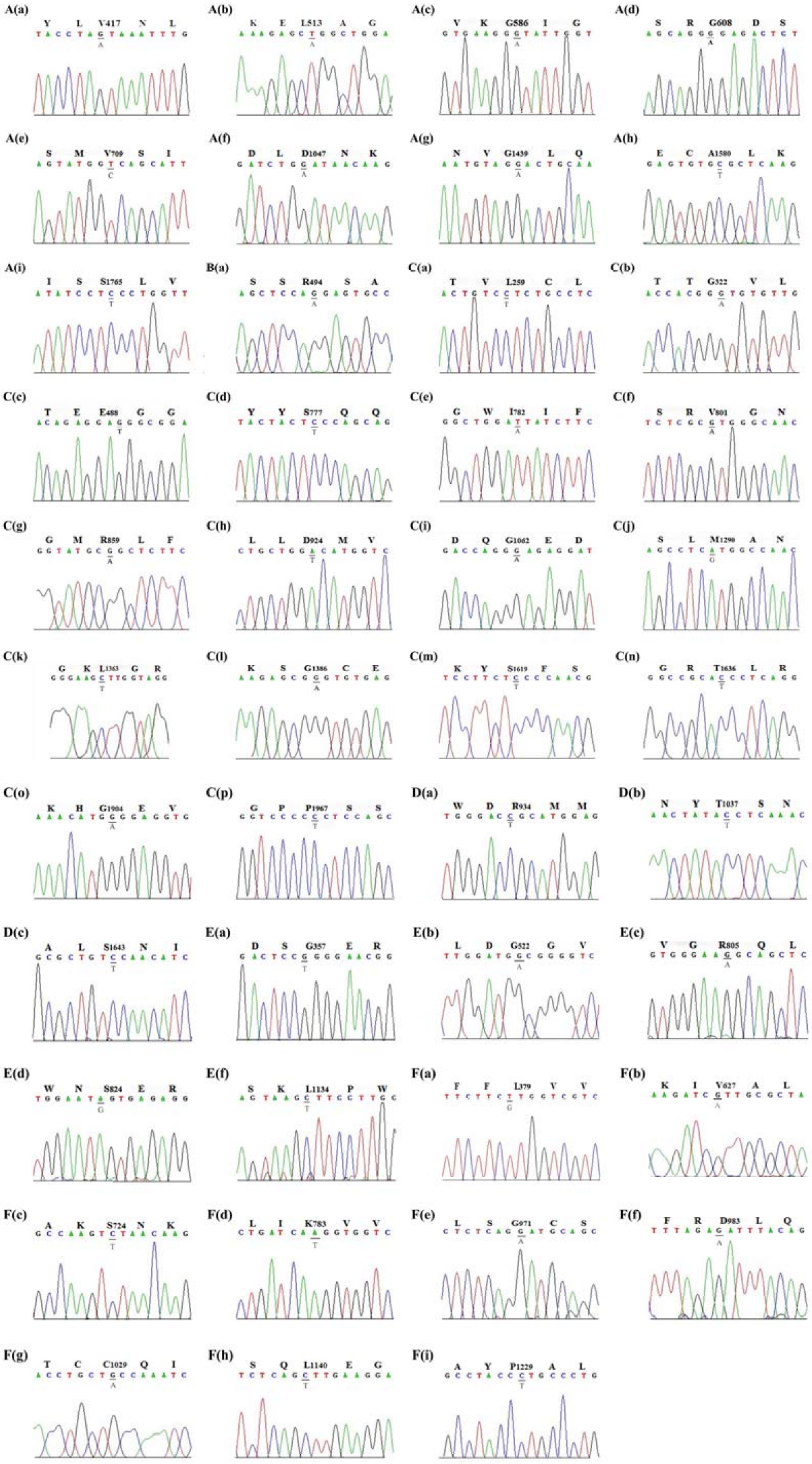
A(a)
A(b)
A(c)
A(d)
A(e)
A(f)
A(g)
A(h)
A(i)
B(a)
C(a)
C(b)
C(c)
C(d)
C(e)
C(f)
C(g)
C(h)
C(i)
C(j)
C(k)
C(l)
C(m)
C(n)
C(o)
C(p)
D(a)
D(b)
D(c)
E(a)
E(b)
E(c)
E(d)
E(f)
F(a)
F(b)
F(c)
F(d)
F(e)
F(f)
F(g)
F(h)
F(i)

Figure S1: RNA editing sites of seven subtypes. Sequence analysis reveals the deduced amino acid sequences and the locations of the editing sites which indicated above the nucleotide sequences. The nucleotide sequences from genomic nucleotides are shown under black line. C_{bm}1.2a: A(d) ,A(e) ,A(h); C_{bm}1.2b :A(a), A(b) ,A(c) ,A(f) ,A(g) ,A(i); C_{bm}1.3a :B(a); C_{bm}1.5a :C(a), C(b), C(f), C(g), C(h) C(k), C(n); C_{bm}1.5b: C(c), C(d), C(e), C(i), C(j), C(l), C(m), C(o), C(p); C_{bm}1.7a: D(a), D(b), D(c); C_{bm}1.7b: D(a), D(b), D(c); C_{bm}1.8a: E(a), E(b), E(c), E(d), E(e); C_{bm}1.8b: E(a), E(b), E(c), E(d), E(e); C_{bm}1.9a: F(a), F(b), F(c), F(d); C_{bm}1.9b: F(a), F(b), F(c), F(d), F(g), F(h), F(i); C_{bm}1.9c: F(a), F(b), F(c), F(d), F(e), F(f).

Table S1: Specific primers for RT-PCR.

All primers were identified by electrophoresis and sequencing of PCR products. *β*-actin and Cav1.3 here were used as internal reference genes and positive control respectively.

Isoform	Primer	Sequence(5'-3')	Location	Product(bp)
$Na_v1.1$(Scn1a)	S	TCAGAGGGAAGCACAGTAGAC	3421-3441	138
	A	TTCCACGCTGATTTGACAGCA	3558-3538	
$Na_v1.2$(Scn2a)	S	ATTTTCGGCTCATTCTTCACACT	4375-4397	176
	A	GGGCGAGGTATCGGTTTTTGT	4550-4530	
$Na_v1.3$(Scn3a)	S	CAGACCATGTGCCTTATTGTGT	2722-2743	154
	A	CCGCGATCTGGAGGTTGTT	2875-2857	
$Na_v1.4$(Scn4a)	S	AGTCCCTGGCAGCCATAGAA	68-87	140
	A	CCCATAGATGAGTGGGAGGTT	207-187	
$Na_v1.5$(Scn5a)	S	GACAATCGTGGGAGCCCTAAT	711-731	148
	A	CTCAGTAAAGTTACGCACGCA	858-838	
$Na_v1.6$(Scn8a)	S	ATGGGGTAGGCTCTCCGAG	1406-1424	156
	A	CCGACTCTGACTTAAACACCTTC	1561-1539	
$Na_v1.7$(Scn9a)	S	TGGATTCCCTTCGTTCACAGA	5534-5554	115
	A	GTCGCAGATACATCCTCTTGTTT	5648-5626	
$Na_v1.8$(Scn10a)	S	TCCGTGGGAACTACCAACTTC	19-39	190
	A	GCTCGCCATAGAACCTGGG	208-190	
$Na_v1.9$(Scn11a)	S	CTGGGGCCTTTTAATCCCATC	334-354	149
	A	GGAATGTTACTGCTAGGACGAC	482-461	
β-actin	S	GGCTGTATTCCCCTCCATCG	84-103	154
	A	CCAGTTGGTAACAATGCCATGT	237-216	
$Ca_v1.3$	S	CAGATCCTGACAGGTGAAGACTGG	2219-2242	314
	A	GTAACCTTGTTGTCACTGTTGGCT	2509-2532	

Table S2: Sequences of primers and their corresponding regions.

Name	Nucleotide sequence	Position	Domain
	Primers for three fragments in middle CDS(Position to mNav1.1[NM_018733.2])		
S1	GAYCCNTGGAACTGGYTRGACTTC	750/773	IS3
AS1	AGGCVADGAABARGTTCAGGACCAC	3100/3124	IIS6
S2	GGCMAAGTCHTGGCCCACMCTGAA	2761/2784	IIS4
AS2	GTGAAGAABSMVCCRAAGATGATGAA	4950/4975	IIIS6
S3	GRGCHTTATCHCGATTTGAAGGVATG	4128/4153	IIIS4
AS3	GTACATGTTNACCACAAYSAGGAAGGA	5471/5497	IVS6
	Primers for full length CDS(Position to each type)		
$Na_v1.1$/S4	TGACAAGATGGAGCAAACAGTGCTTG	-7/19	
$Na_v1.1$/AS4	ATTTCCCTTTGGCTTTTTCATCYTTBC	5969/5995	
$Na_v1.2$/S4	AAGATGGCACRRTCAGTGCTGGTAC	-3/22	
$Na_v1.2$/AS4	ACTTTTTACTTTCCCTGATATCTTTCCC	5992/6019	
$Na_v1.3$/S4	ATGGCCCAGGCCCTGCTGGTGC	1/22	
$Na_v1.3$/AS4	CACCTCCTTGCCCTTGMTCTCCTTC	5817/5841	
$Na_v1.4$/S4	GATGGCCAGMTCATCTCTGCCCAC	-1/23	Span
$Na_v1.4$/AS4	AGACAAGAGACTCTTTGACCCCTGGG	5499/5524	ATG/
$Na_v1.5$/S4	ATGGCCAACYTSCTGCTGCCCAGGG	1/25	TGA
$Na_v1.5$/AS4	GAGGTTCACACTATAGACTCTCGGTC	6046/6070	
$Na_v1.6$/S4	ATGGCCGCCAGGCTGCTGGCC	1/21	
$Na_v1.6$/AS4	GCACTTGCTCTCCCTCACCTCCTTCT	5909/5934	
$Na_v1.7$/S4	ATGGCGATGYTGCCTCCBCCAG	1/22	
$Na_v1.7$/AS4	GCTCTATTTCCTGCTTTCGRTCTTTCTCT	5903/5931	
$Na_v1.8$/S4	AAGATGGAGYTCCCCWTTGGGTCCGT	-3/23	
$Na_v1.8$/AS4	TCACTGAGGTCCAGGGCTSTTYCCTT	58512/5877	
$Na_v1.9$ S4	AGGGTGAAGATGGAKGASAGGTRCTACC	-9/19	
$Na_v1.9$/AS4	GGTGGGGGTTTCAGTCACAATGAACC	5335/5360	

Three pairs of degenerate primers derived from conserved region of mammalian VGSCs (IS3, IIS6, IIS4, IIIS6, IIIS4 and IVS6) were used for cloning three fragments in middle CDS. Nine pairs of specific primers were designed spanning initiation codon and termination codon to clone the coding sequences (CDS) of all the VGSCs.

Materials and Methods

Tissue preparation

Acutely dissected cochlear sensory epithelia (n=100) of C57BL6 mice (Laboratory Animal Centre, Fudan University, China) were studied in from postnatal day 3 to 9. The cochleae were dissected in extracellular solution composed of (mM): 135 NaCl, 5.8 KCl, 1.3 $CaCl_2$, 0.9 $MgCl_2$, 0.7 NaH_2PO_4, 5.6 D-glucose, 10 Hepes-NaOH, 2 Sodium pyruvate. The pH was adjusted to 7.5 and the osmolality was about 308 mmol kg^{-1}. Carefully remove the stria vascularis, vestibular membrane , spiral ganglions and tectorial membrane connected to basilar membrane by fine forceps, and then use microelectrode to detach the sensory epithelia that clustered with hair cells. The investigation was approved by the Ethic Committee and the Committee of Animal Experimentation of Shanghai University. All efforts were made to minimize the number of animals used and their suffering.

RNA extraction and RT PCR

Total RNA was respectively extracted from the cochlear sensory epithelia of mice by Trizol reagent (Invitrogen, USA) according to manufacturer's protocol. RNA integrity was confirmed by the Agilent 2100 Bioanalyzer (Agilent Technologies) with clear characteristic peaks at 28S and 18S. First-strand cDNA was synthesized from total RNA by using oligo$(dT)_{18}$ and PrimeScriptRTase (Takara, Japan).

Quantitative real-time PCR

The quantity of cDNA was measured by using a spectrophotometer prior to $_q$RT- PCR, which was performed with the SYBR Green master mix Kit and Bio-Rad Real Time PCR system. Each qRT-PCR reaction (25μl final volume) contained 1x SYBR Green master mix, 1μl of cDNA, and a sodium channel transcript specific primer pair designed according to each of the sequences (Table S1). All samples, including the ’no-template’ negative control, were performed in triplicate. The reaction cycle consisted of a melting step of 50ºC for 2 min then 95℃ for 10 min, followed by 40 cycles of 95℃ for 10 sec and 60℃ for 45 sec. Specificity of the PCR reactions was assessed via a melting curve analysis for each PCR reaction using CFX Manager software. Relative expression levels for the sodium channel transcripts were calculated by the $2^{-\Delta\Delta CT}$ method. The β-actin RNA, an endogenous control, was used to normalize the expression of targets. Each experiment was repeated three to four times with different preparations of RNA samples. Statistical analyses were performed using the least significant difference (LSD) test at P=0.01 and P=0.05 with DPS statistical software.

Amplification of the full length of VGSC transcripts in the sensory epithelia

Degenerate primers derived from conserved amino acid residues of mammalian voltage-gated sodium channels, were used for amplification of the homologous sequence from sensory epithelia cDNA. Sequence of the primers and their corresponding position are listed (Table S2). PCR amplification was carried out with Advantage high-fidelity polymerase (Takara, Japan) based on the Veriti Thermal

Cycler (Gene, USA). The PCR products were analyzed on agarose gels and purified for direct sequencing. Finally, they were sequenced on an ABI PRISM 377 DNA sequencer (Perkin–Elmer). Cloning and sequence analysis of sodium channel cDNA fragments were repeated at least three times for each fragment with different preparations of total RNA
